# Effect of blood flow–restricted resistance training on myocardial fibrosis in early spontaneously hypertensive rats

**DOI:** 10.3389/fcvm.2023.1101748

**Published:** 2023-02-01

**Authors:** Zhaowen Tan, Peiyou Chen, Yuchan Zheng, Ying Pan, Baolong Wang, Yan Zhao

**Affiliations:** ^1^School of Sports Science and Physical Education, Nanjing Normal University, Nanjing, Jiangsu, China; ^2^Nanjing Sport Institute, Nanjing, Jiangsu, China

**Keywords:** blood flow-restricted resistance training, early spontaneous hypertension, myocardial fibrosis, TGFβ-1-Smad, MMPs-TIMPs

## Abstract

**Objective:**

The purpose of this study was to explore the effect of blood flow–restricted resistance training on myocardial fibrosis in early spontaneously hypertensive rats (SHRs).

**Methods:**

Four-week-old male Wistar-Kyoto rats and SHRs were randomly divided into the following groups: normal group (WKY), SHR control (SHR-SED) group, high-intensity resistance training (HIRT) group, low- and medium-intensity resistance training (LMIRT) group, and blood flow–restricted low- and medium-resistance training (BFRT) group. Body weight, hemodynamics, cardiac function, myocardial morphology and fibrosis, and the expression levels of transforming growth factor-beta1-Smad (TGFβ-1-Smad) pathway-related proteins in the myocardium were assessed.

**Results:**

(1) BFRT lowered blood pressure significantly, decreased left ventricular wall thickness, and improved cardiac function. At the same time, BFRT was superior to traditional resistance training in lowering diastolic blood pressure, and was superior to HIRT in improving left ventricular compliance, reducing heart rate, and reducing left ventricular posterior wall and left ventricular mass (*P* < 0.05). (2) BFRT decreased collagen I and collagen fiber area in the myocardium, increased the collagen III area, and decreased the collagen I/III ratio (*P* < 0.05). BFRT produced a better proportion of myocardial collagen fibers than did traditional resistance training (*P* < 0.05). (3) In the myocardium of the BFRT group compared to the traditional resistance training group, the expression of TGFβ-1, Smad2/3/4, p-Smad2/3, CTGF, and TIMP1 was significantly downregulated, MMP2 and TIMP2 were significantly upregulated, the MMP/TIMP ratio significantly increased, and TGFβ-1 expression significantly decreased (*P* < 0.05).

**Conclusion:**

BFRT inhibited the TGFβ-1-Smad pathway in the myocardium, downregulated the expression of CTGF, and regulated the balance between MMPs and TIMPs, thereby reducing myocardial fibrosis in SHR, and improving cardiac morphology and function. BFRT also lowered blood pressure, and achieved an effect of early prevention and treatment of hypertension. At the same time, BFRT was superior to traditional resistance training in reducing diastolic blood pressure and adjusting the proportion of myocardial collagen fibers.

## 1. Introduction

As a high-risk factor for cardiovascular disease, hypertension leads to the enhancement of physiological and neurohumoral signals and an increased hemodynamic load in the process of disease development, gradually causing cardiovascular fibrosis, structural remodeling, and diastolic dysfunction. Over time, hypertension eventually causes end organ damage (1, 2).

According to a research report on hypertension treatment, exercise therapy can effectively reduce blood pressure and improve cardiovascular function (3–5), but this treatment for advanced hypertension is not very successful. The adaptive increase in the thickness of the heart and blood vessel walls can no longer be fully normalized through pressure reduction after exercise intervention, because the longer the hypertension lasts, the older the individual becomes. This leads to more fibrous tissue in the myocardium and connective tissue in the blood vessel walls, and increasingly irreversible fibrotic structural changes. In this regard, treatment intervention should be carried out at an early stage when the adaptive changes in the cardiovascular structure are still minor. The purpose of antihypertensive treatment is not only to reduce blood pressure but also to reverse the structural changes caused by hypertension (6, 7). Therefore, it is necessary for us to carry out early prevention and treatment of hypertension, and it is very important to reduce hypertensive myocardial fibrosis. The transforming growth factor-beta1-Smad (TGFβ-1-Smad) signal pathway is an important pathway that promotes myocardial fibrosis. When this pathway is activated, Smad2/3 is phosphorylated in large quantities, and combines with Smad4 to form a transcriptional complex, which mediates the transmission of subsequent signaling pathways and induces an increase in connective tissue growth factor (CTGF) expression levels. At the same time, it adjusts the balance between matrix metalloproteinases (MMPs) and tissue inhibitor of matrix metalloproteinases (TIMPs), promotes the production and proliferation of the extracellular matrix, and leads to the development of myocardial interstitial fibrosis (8). Some studies have shown that exercise training reduced myocardial fibrosis by downregulating the expression of TGFβ-1-Smad pathway-related proteins (9). The balance between MMPs and TIMPs also mediates the degradation and synthesis of collagen fibers (10), and therefore is one of the targets of myocardial fibrosis treatment.

Blood flow-restriction (BFR) resistance training is a new resistance training method that has emerged in recent years and has been found to be safe and feasible (11, 12). In a study of the acute effects of hypertension, the immediate effect of low-intensity BFR resistance training on the reduction of systolic blood pressure of hypertensive women after exercise was better than that of medium-intensity resistance training without BFR. Low-intensity exercise combined with BFR is considered as a non-drug intervention for blood pressure control of hypertensive patients (13, 14). Some studies have shown that blood flow–restricted resistance training improved the heart function of elderly rats and had a positive effect on blood pressure control (15). In a long-term intervention study of hypertension, it was found that blood flow–restricted training (BFRT) lowered blood pressure and improved cardiac autonomic nervous function in elderly hypertensive patients (16). The above studies confirmed the effect of BFR exercise on lowering blood pressure and improving cardiac function in elderly and mature hypertensive animals and humans. However, the mechanism of how BFRT causes blood pressure changes and cardiac function improvement is still unclear. Furthermore, in the early prevention and treatment of hypertension, whether blood flow restriction, combined with medium and low-intensity resistance training, can improve myocardial fibrosis and the underlying mechanism needs further research.

Therefore, this study aimed to explore the effect of blood flow-restriction, combined with low and medium-intensity resistance training, on myocardial fibrosis in early spontaneously hypertensive rats, and explored its possible mechanism.

## 2. Materials and methods

### 2.1. Animals

Sixty clean-grade male spontaneously hypertensive rats (SHR) and 15 clean-grade male Wistar-Kyoto rats (4 weeks old) weighing approximately 200 g were obtained from Qinglongshan Animal Feeding Base in Jiangning District, Nanjing, China. The animals were housed in a 20–23°C environment in a 12-h light/12-h dark cycle with free access to water and food. The study was approved by the Animal Experiment Ethics Committee of the Nanjing Sport Institute.

### 2.2. Grouping of rats

After 1 week of adaptive feeding, all rats were randomly divided into five groups: WKY (Wistar-Kyoto, *n* = 15), SHR-SED (SHR controls, *n* = 15), HIRT (high-intensity resistance training, *n* = 15), LMIRT (low and medium-intensity resistance training, *n* = 15), and BFRT (blood flow restriction combined with low and medium-intensity resistance training, *n* = 15).

### 2.3. Exercise plan

The exercise groups trained on a ladder suitable for rats. Before the maximum load test, all animals became accustomed to climbing the ladder for two consecutive weeks. The maximum load test included an initial load that was 75% of the body weight, and was attached to the base of the rat’s tail. The load was gradually increased by 50 g during subsequent climbs. During formal training, the standardized maximum load of each rat (the load of the last complete climb) was used for resistance training and adjusted according to the animal’s weight each week. The resistance training mode was a gradually increasing load (the exercise groups received exercise intervention for 8 weeks starting at the age of 7 weeks) (17). The specific exercise plan is detailed in a previous study of the project team (18).

The blood flow restriction combined with low and medium-intensity resistance training was as follows: 5 days a week; weeks 1–2: 30–40% of the maximum load; weeks 3–5: 40–50% of the maximum load; and weeks 6–8: 40–60% of the maximum load. Additionally, 30–40% blood flow restriction was performed at the same time as ladder resistance training. The rubber band was used to bind the root of the thigh of the right lower limb of the rats to apply vascular blood flow limitation with resistance training. At 1-min intervals, blood flow restriction was lifted, and blood flow was reperfused. After rest, the rubber band was used to bind the site again (18).

The low and medium-intensity resistance training was as follows: 5 days a week; weeks 1–2: 30–40% of the maximum load; weeks 3–5: 40–50% of the maximum load; and weeks 6–8: 40–60% of the maximum load.

The high-intensity resistance training was as follows: 5 days a week; weeks 1–2: 50–60% of the maximum load; weeks 3–5: 60–70% of the maximum load; and weeks 6–8: 70–80% of the maximum load.

### 2.4. Measurement of blood pressure and heart rate

Non-invasive blood pressure measurements of rat tail arteries were measured at Jiangsu Medical Animal Experimental Center. During the test, the environment was quiet, warm, and appropriate, and the rats were kept awake. The caudal artery blood pressure (BP) and heart rate (HR) of the rats at rest were measured using an intelligent non-invasive blood pressure tester BP-2000 (Ruanlong Biological Co., Beijing, China). Each rat was measured continuously three times, and the average value was used (18).

### 2.5. Echocardiography

The hearts of the rats were examined by echocardiography at the Jiangsu Medical Animal Experimental Center. After applying a 2–3% isoflurane anesthesia to the rats, a small animal high-frequency color ultrasound (Visual Sonics, Canada) was used to evaluate cardiac function in the five groups, including ejection fraction (EF), fractional shortening (FS), E peak/A peak of the mitral valve (MV E/A), interventricular septal thickness at end-diastole (IVSd), interventricular septal thickness at end-systole (IVSs), left ventricular internal diameter at end-diastole (LVIDd), left ventricular internal diameter at end-systole (LVIDs), left ventricular posterior wall end-diastolic thickness (LVPWd), left ventricular posterior wall end-systolic thickness (LVPWs), and left ventricular mass (LV mass). All rats were measured three times (18).

### 2.6. Hematoxylin and eosin staining and sirius red staining

The heart tissue was fixed with paraformaldehyde for 48 h and then used for histological staining. After being washed with water, heart samples were dehydrated, cleared until transparent, embedded in paraffin, and cut into 5-μm-thick microtome sections. Hematoxylin and eosin staining (HE) and sirius red staining (SR) were performed on myocardial tissue sections to observe the myocardial morphology and evaluate the degree of myocardial fibrosis. The left ventricular wall thickness (LVWT), the total area of myocardial fibers, the number of myocardial fibers, the area of a single myocardial fiber, and the percentage of collagen I and III were analyzed by Image-Pro Plus 6.0 software [The percentage of collagen I and III (%) = The area of collagen I and III/Tissue area*100%] (8, 9).

### 2.7. Western blotting

The left ventricular tissues of rats were removed and ground into powder in liquid nitrogen, and then incubated with a cell lysis solution for 30 min. Then, the solution was centrifuged at 10,000 rpm at 4°C for 5 min, and the supernatant was collected. The total protein concentration of the solution was determined using the BCA protein quantitative method. Equal amounts of protein (50 μg/well) were separated on 10% SDS-polyacrylamide gels and transferred onto a PVDF membrane (Bio-Rad Laboratories, USA). The membranes were blocked with 3% non-fat milk solution in Tris-buffered saline (TBS) with 0.1% Tween 20 (TBS-T) for 1.5 h, then incubated overnight at 4°C with monoclonal primary antibodies (anti-TGFβ-1, 1:2,000, Cell Signaling, USA; anti-Smad2/3/4, 1:2,000; anti-TIMP2/9, 1:2,000, Affinity Biosciences, China; anti-p-Smad2/3, 1:1,000; anti-MMP2/9, 1:2,000, Immunoway Biotechnology, China; anti-GAPDH, 1:50,000; anti-β-actin, 1:50,000, Proteintech Group, USA). After washing for 30 min (3 washes of 10 min) in TBS-T, the membranes were incubated with a polyclonal peroxidase-conjugated secondary antibody (anti-rabbit IgG-HRP, 1:1,000, Proteintech Group) and incubated at room temperature for 1.5 h. Finally, an enhanced chemiluminescence reagent was added, and the membranes were placed in a Bio-Rad ChemiDoc XRS + (Bio-Rad, USA) for exposure. The grayscale was analyzed with the image analysis software Image Lab (19).

### 2.8. Statistical analysis

SPSS 20.0 ANOVA was used for inter-group comparisons and paired *t*-tests were used to analyze differences within groups. The statistical data were presented as mean and standard deviation, and the effect size and 95% CI of the mean differences were calculated. Statistical significance was established at *P* ≤ 0.05. Image Lab was used to analyze the results of western blotting, and GraphPad Prism 8 was used to generate the statistical chart.

## 3. Results

### 3.1. Bodyweight (BW), heart weight (HW), hemodynamic parameters, and cardiac function

**FIGURE 1:**
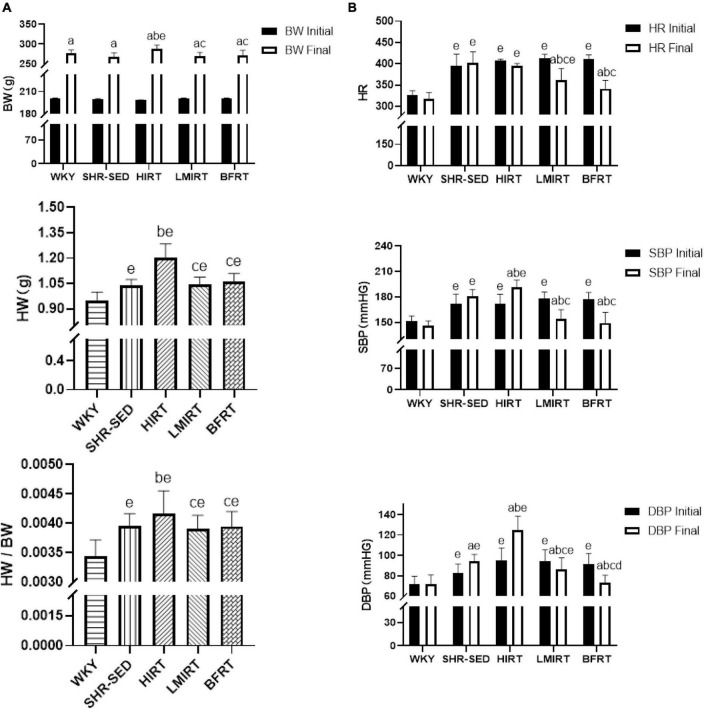
**(A)** Body weight, heart weight, and heart weight/body weight; **(B)** heart rate and blood pressure. The values are mean ± *SD*. *n* = 12 rats in each group. BW, body weight; HW, heart weight; SBP, systolic blood pressure; DBP, diastolic blood pressure; HR, heart rate. *^a^p* < 0.05 vs. Before training; *^b^p* < 0.05 vs. SHR–SED; *^c^p* < 0.05 vs. HIRT; *^d^p* < 0.05 vs. LIRT; *^e^p* < 0.05 vs. WKY.

Body weight and heart weight findings showed that: (1) Before training, the bodyweight of the SHRs in the five groups showed no significant difference (*P* < 0.05). (2) After training, the bodyweight of the five groups significantly increased (*P* < 0.05). (3) After training, compared with the WKY group, the HW and HW/BW ratio in each group increased significantly, among which the HW (95% CI of diff: −0.325 to −0.190, *P* = 0.001) and HW/BW (95% CI of diff: −0.001 to −0.001, *P* = 0.001) in the HIRT group increased most significantly, and there was a significant difference with the other groups (*P* < 0.05) (Table 1 and Figure 1A).

**TABLE 1:** Bodyweight, heart weight, hemodynamic parameters, and cardiac function.

	*F*	df	η^2^	*P*-value
BW initial	1.257	4,60	0.067	0.295
BW final	3.130	4,60	0.152	0.020
HR initial	27.461	4,55	0.611	0.000
HR final	14.673	4,55	0.456	0.000
SBP initial	6.387	4,55	0.267	0.000
SBP final	22.574	4,55	0.563	0.000
DBP initial	4.359	4,55	0.199	0.003
DBP final	52.307	4,55	0.792	0.000
HW	14.995	4,50	0.545	0.000
HW/BW	15.643	4,50	0.556	0.000
EF initial	8.073	4,30	0.466	0.000
EF final	13.239	4,30	0.589	0.000
FS initial	7.919	4,30	0.461	0.000
FS final	11.332	4,30	0.551	0.000
MV E/A initial	4.419	4,30	0.336	0.005
MV E/A final	25.367	4,30	0.744	0.000
LV mass initial	4.830	4,30	0.356	0.003
LV mass final	20.051	4,30	0.696	0.000
IVSd initial	0.385	4,30	0.042	0.818
IVSd final	6.364	4,30	0.421	0.001
IVSs initial	0.511	4,30	0.055	0.728
IVSs final	4.106	4,30	0.319	0.008
LVIDd initial	3.557	4,30	0.278	0.015
LVIDd final	6.300	4,30	0.405	0.001
LVIDs initial	7.707	4,30	0.454	0.000
LVIDs final	14.732	4,30	0.614	0.000
LVPWd initial	0.224	4,30	0.025	0.923
LVPWd final	5.838	4,30	0.400	0.001
LVPWs initial	3.948	4,30	0.311	0.010
LVPWs final	3.921	4,30	0.309	0.010

Hemodynamic findings showed that: (1) Compared with the SHR-SED group, BFRT and LMIRT effectively lowered the BP of SHRs (*P* < 0.05), and BFRT had a better effect on lowering the diastolic BP (*P* < 0.05). (2) Compared with SHR-SED, HIRT significantly increased the BP of SHRs (*P* < 0.05), and the diastolic BP increased significantly (95% CI of diff: 4.856–28.512, *P* = 0.007) (Table 1 and Figure 1B).

**FIGURE 2:**
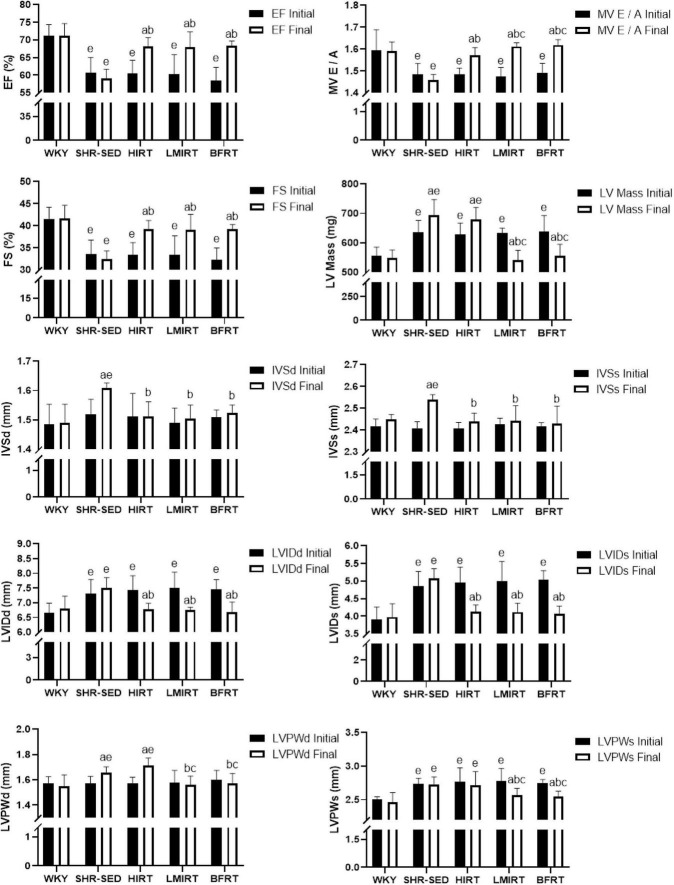
Cardiac function. The values are mean ± *SD*. *n* = 8 rats in each group. EF, ejection fraction; FS, fraction shortening; MV E/A, E peak/A peak of mitral valve; LV Mass, left ventricular mass; IVSd, interventricular septal thickness at end–diastole; IVSs, interventricular septal thickness at end–systole; LVIDd, left ventricular internal diameter at end–diastole; LVIDs, left ventricular internal diameter at end–systole; LVPWd, left ventricular posterior wall end–diastolic thickness; LVPWs, left ventricular posterior wall end– systolic thickness. *^a^p* < 0.05 vs. Before training; *^b^p* < 0.05 vs. SHR–SED; *^c^p* < 0.05 vs. HIRT; *^e^p* < 0.05 vs. WKY.

Echocardiography findings showed that: (1) Compared with the SHR-SED group, BFRT and LMIRT improved the EF, FS, and MV E/A, reduced the HR, IVSd, IVSs, LVIDd, LIVDs, LVPWd, LVPWs, and LV mass in SHRs (*P* < 0.05). (2) Compared with the SHR-SED group, HIRT improved the EF, FS, and MV E/A of SHRs and decreased the IVSd, IVSs, LVIDd, and LIVDs (*P* < 0.05), but did not decrease the HR, LVPW, or LV mass (*P* > 0.05). (3) BFRT and LMIRT were superior to HIRT in improving left ventricular compliance (BFRT: 95% CI of diff: −0.084 to −0.010, *P* = 0.015; LMIRT: 95% CI of diff: −0.078 to −0.004, *P* = 0.033), reducing HR (BFRT: 95% CI of diff: 27.974–80.582, *P* = 0.001; LMIRT: 95% CI of diff: 7.347–59.951, *P* = 0.013), LVPW (BFRT LVPWd: 95% CI of diff: 0.056–0.228, *P* = 0.002, LVPWs: 95% CI of diff: 0.002–0.330, *P* = 0.047; LMIRT LVPWd: 95% CI of diff: 0.069–0.241, *P* = 0.001), and LV mass (BFRT: 95% CI of diff: 74.232–171.243, *P* = 0.001; LMIRT: 95% CI of diff: 89.794–186.826, *P* = 0.001) (Table 1 and Figures 1B, 2).

### 3.2. Morphology and structure of myocardium

**FIGURE 3:**
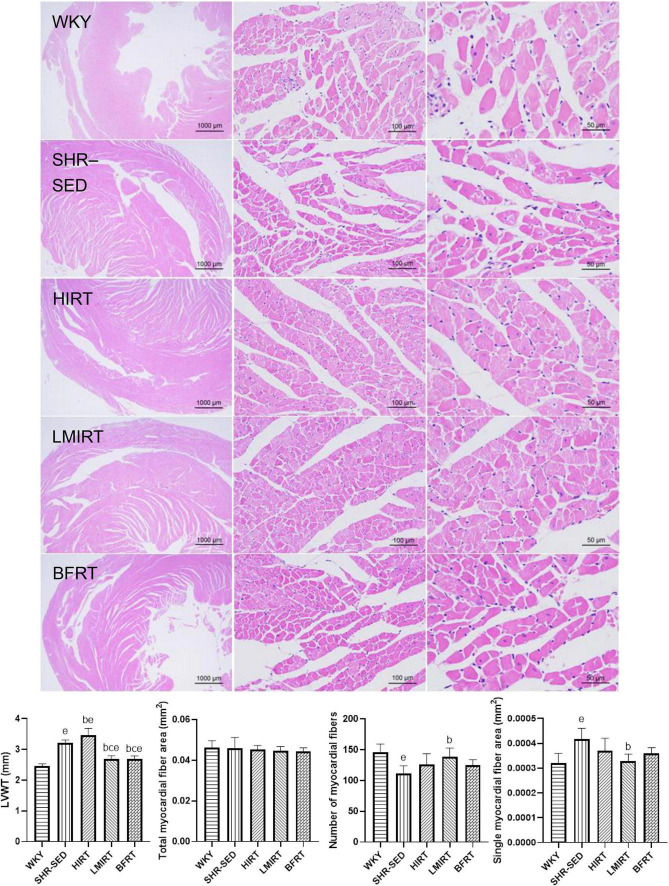
Hematoxylin–eosin staining of left ventricular tissues. The values are mean ± *SD*. *n* = 8 rats in each group. LVWT, left ventricular wall thickness. *^b^p* < 0.05 vs. SHR–SED; *^c^p* < 0.05 vs. HIRT; *^e^p* < 0.05 vs. WKY.

HE staining findings showed that: (1) Compared with the WKY group, the myocardial cells of SHR-SED rats were enlarged, the intercellular space was enlarged, and the arrangement of myocardial fibers was blurred. In the exercise groups, the myocardial cells were not enlarged, and the myocardial fibers were arranged clearly. (2) Through the analysis and calculations of Image-Pro Plus 6.0, compared with the SHR-SED group, LMIRT and BFRT reduced the LVWT (BFRT: 95% CI of diff: 0.349–0.682, *P* = 0.001; LMIRT: 95% CI of diff: 0.344–0.678, *P* = 0.001), HIRT increased the LVWT (95% CI of diff: −0.420 to −0.086, *P* = 0.004), and LMIRT significantly increased the myocardial cell density (95% CI of diff: 4.218e-5 to 13.82 e-5, *P* = 0.001) (Table 2 and Figure 3).

**TABLE 2:** Hematoxylin–eosin staining and sirius red staining of left ventricular tissues.

	*F*	df	η^2^	*P*-value
LVWT	50.406	4,35	0.818	0.000
Total myocardial fiber area	0.282	4,35	0.024	0.888
Number of myocardial fibers	4.841	4,35	0.301	0.002
Single myocardial fiber area	5.268	4,35	0.319	0.001
Collagen I%	76.697	4,35	0.872	0.000
Collagen III%	6.759	4,35	0.375	0.000
Collagen I/III	42.875	4,35	0.792	0.000
Collagen area/total area	65.250	4,35	0.853	0.000

### 3.3. Degree of myocardial fibrosis

**FIGURE 4:**
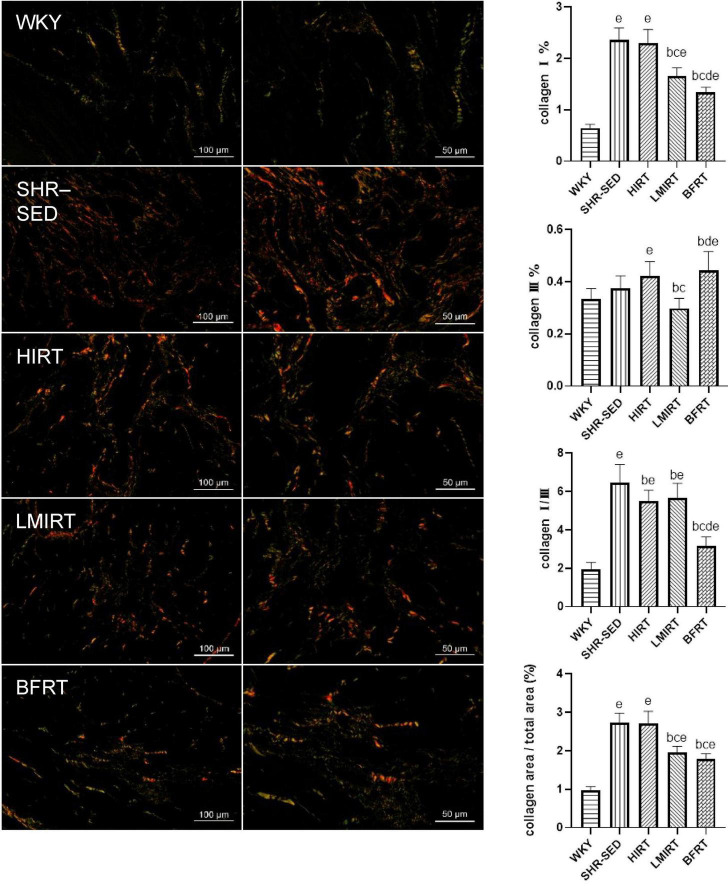
Sirius red staining of left ventricular tissues. Under polarized light, collagen I was red or yellow with strong double refraction; collagen III is green and weakly birefringent. The values are mean ± *SD*. *n* = 8 rats in each group. *^b^p* < 0.05 vs. SHR–SED; *^c^p* < 0.05 vs. HIRT; *^d^p* < 0.05 vs. LIRT; *^e^p* < 0.05 vs. WKY.

Sirius red staining findings showed that, compared with the SHR-SED group, BFRT decreased the area of collagen I and collagen fibers in the myocardium, increased the area of collagen III, and decreased the ratio of collagen I/III (collagen I%: 95% CI of diff: 0.788–1.251, *P* = 0.001; collagen III%: 95% CI of diff: −0.134 to −0.002, *P* = 0.043; collagen I/III: 95% CI of diff: 2.723–4.331, *P* = 0.001; collagen area/total area: 95% CI of diff: 0.693–1.209, *P* = 0.001). LMIRT also decreased the collagen I and collagen III in the myocardium, and decreased the ratio of the collagen fiber area to the collagen I/III area (collagen I%: 95% CI of diff: 0.477–0.941, *P* = 0.001; collagen III%: 95% CI of diff: 0.0118–0.143, *P* = 0.022; collagen I/III: 95% CI of diff: 0.1991–1.807, *P* = 0.016; collagen area/total area: 95% CI of diff: 0.528–1.044, *P* = 0.001). Although HIRT decreased the ratio of collagen I/III (95% CI of diff: 0.357–1.964, *P* = 0.006), it had no significant effect on collagen I and III in the myocardium (collagen I%: 95% CI of diff: −0.164–0.299, *P* = 0.560; collagen III%: 95% CI of diff: −0.112 to 0.020, *P* = 0.166). Furthermore, compared with traditional resistance training, BFRT showed a better adjustment of the proportion of myocardial collagen fibers (95% CI of diff: 1.741–3.306, *P* = 0.001) (Table 2 and Figure 4).

### 3.4. TGFβ-1-Smad pathway-related protein expression in the myocardium

**FIGURE 5:**
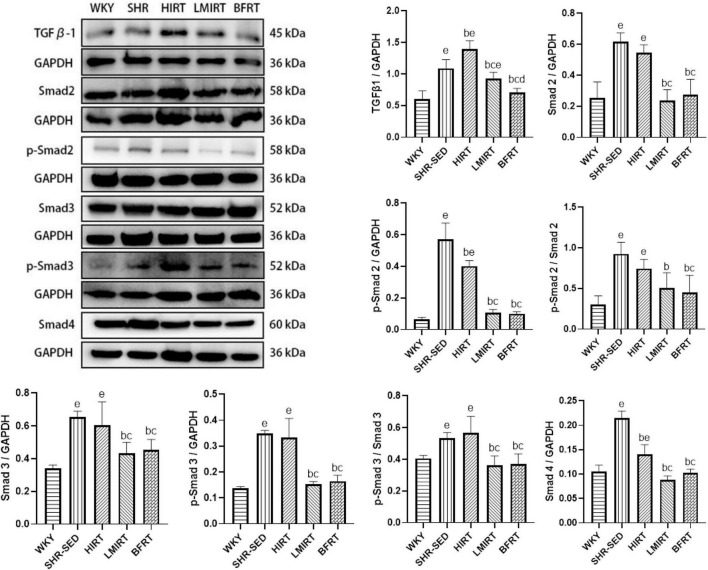
TGF β–1–Smad protein expression in left ventricular tissues. The values are mean ± *SD*. *n* = 8 rats in each group. TGF β–1, transforming growth factor–β1; GAPDH, glyceraldehyde–3–phosphate dehydrogenase. *^b^p* < 0.05 vs. SHR–SED; *^c^p* < 0.05 vs. HIRT; *^d^p* < 0.05 vs. LIRT; *^e^p* < 0.05 vs. WKY.

Western blot analysis showed that, compared with the SHR-SED group, BFRT and LMIRT inhibited the TGFβ-1-Smad signal pathway in the myocardium of SHRs, decreased the TGFβ-1 expression level, and reduced the phosphorylation of Smad2/3 (BFRT: TGFβ-1: 95% CI of diff: 0.246–0.525, *P* = 0.001, p-Smad2/Smad2: 95% CI of diff: 0.283–0.668, *P* = 0.001, p-Smad3/Smad3: 95% CI of diff: 0.086–0.242, *P* = 0.001; LMIRT: TGFβ-1: 95% CI of diff: 0.024–0.303, *P* = 0.023, p-Smad2/Smad2: 95% CI of diff: 0.230–0.614, *P* = 0.001, p-Smad3/Smad3: 95% CI of diff: 0.094–0.249, *P* = 0.001). Although HIRT decreased the expression levels of p-Smad2 and Smad4, it increased TGFβ-1 expression (TGFβ-1: 95% CI of diff: −0.452 to −0.173, *P* = 0.001; p-Smad2: 95% CI of diff: 0.107–0.229, *P* = 0.001; Smad4: 95% CI of diff: 0.058–0.090, *P* = 0.001), the overall level of phosphorylation activation of Smad2/3 did not change, and the TGFβ-1-Smad signal pathway was activated. Compared with LMIRT, BFRT decreased TGFβ-1 expression more significantly (95% CI of diff: 0.082–0.361, *P* = 0.003) (Table 3 and Figure 5).

**TABLE 3:** TGF β–1–Smad, MMPs, TIMPs, and CTGF protein expression in left ventricular tissues.

	*F*	df	η^2^	*P*-value
TGFβ-1	42.497	4,35	0.829	0.000
p-Smad2	111.821	4,35	0.927	0.000
Smad2	29.909	4,35	0.774	0.000
p-Smad2/Smad2	13.702	4,35	0.610	0.000
p-Smad3	47.725	4,35	0.845	0.000
Smad3	15.644	4,35	0.641	0.000
p-Smad3/Smad3	12.444	4,35	0.587	0.000
Smad4	86.289	4,35	0.908	0.000
MMP2	37.858	4,35	0.812	0.000
MMP9	23.615	4,35	0.730	0.000
TIMP2	279.761	4,35	0.970	0.000
TIMP1	753.566	4,35	0.989	0.000
MMP2/TIMP2	35.555	4,35	0.803	0.000
MMP9/TIMP1	94.859	4,35	0.916	0.000
CTGF	26.815	4,35	0.754	0.000

**FIGURE 6:**
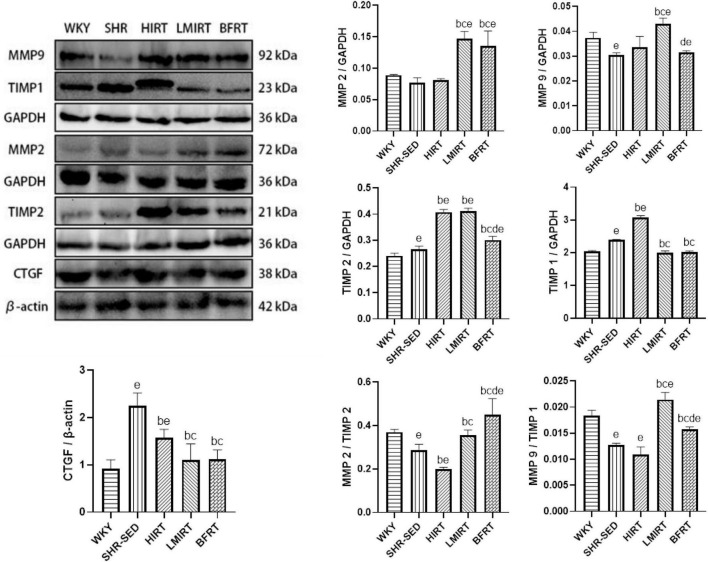
MMPs, TIMPs and CTGF protein expression in left ventricular tissues. The values are mean ± *SD*. *n* = 8 rats in each group. MMPs, matrix metalloproteinases; TIMPs, tissue inhibitor of matrix metalloproteinases; CTGF, connective tissue growth factor; GAPDH, glyceraldehyde–3–phosphate dehydrogenase; β–actin, beta actin. *^b^p* < 0.05 vs. SHR–SED; *^c^p* < 0.05 vs. HIRT; *^d^p* < 0.05 vs. LIRT; *^e^p* < 0.05 vs. WKY.

Compared with the SHR-SED group, BFRT increased the expression of MMP2, and TIMP2, decreased the expression of TIMP1, adjusted the balance between MMPs and TIMPs, and increased the proportion of MMPs in the MMP/TIMP ratio (MMP2: 95% CI of diff: −0.074 to −0.043, *P* = 0.001; TIMP2: −0.048 to −0.021, *P* = 0.001; TIMP1: 0.337–0.434, *P* = 0.001; MMP2/TIMP2: −0.207 to −0.116, *P* = 0.001; MMP9/TIMP1: −0.004 to −0.002, *P* = 0.001). LMIRT increased the expression of MMP2, MMP9, and TIMP2, and decreased the expression of TIMP1, and also adjusted the balance between MMPs and TIMPs, and increased the proportion of MMPs in the MMP/TIMP ratio (MMP2: 95% CI of diff: −0.085 to −0.055, *P* = 0.001; MMP9: −0.015 to −0.010, *P* = 0.001; TIMP2: −0.159 to −0.132, *P* = 0.001; TIMP1: 0.342–0.438, *P* = 0.001; MMP2/TIMP2: −0.114 to −0.023, *P* = 0.004; MMP9/TIMP1: −0.010 to −0.007, *P* = 0.001). HIRT only upregulated the expression of TIMP1 and TIMP2, and the ratio of MMP2 to TIMP2 with HIRT decreased (TIMP1: −0.729 to −0.633, *P* = 0.001; TIMP2: −0.156 to −0.128, *P* = 0.001; MMP2/TIMP2: 0.044–0.135, *P* = 0.001). In the balance between MMPs and TIMPs, BFRT was better able to increase the ratio of MMP2/TIMP2, and LMIRT was better able to increase the ratio of MMP9/TIMP1 (MMP2/TIMP2: −0.138 to −0.048, *P* = 0.001; MMP9/TIMP1: 0.004–0.007, *P* = 0.001). Compared with the SHR-SED group, the expression of CTGF in the exercise groups was downregulated (HIRT: 95% CI of diff: 0.371–0.964, *P* = 0.001; LMIRT: 95% CI of diff: 0.842–1.435, *P* = 0.001; BFRT: 95% CI of diff: 0.830–1.424, *P* = 0.001), and was downregulated the most in the BFRT and LMIRT groups (LMIRT: 95% CI of diff: 0.174–0.768, *P* = 0.003; BFRT: 95% CI of diff: 0.163–0.756, *P* = 0.003), with no significant difference between the two groups (95% CI of diff: −0.308 to 0.285, *P* = 0.939) (Table 3 and Figure 6).

## 4. Discussion

The progress and characteristics of hypertension in SHRs are quite similar to those of human primary hypertension. At the age of 5–10 weeks in SHR, the blood pressure starts to rise rapidly (6, 20), and the cardiovascular system subsequently appears to functionally compensate. At the same time, collagen deposition and fibrosis occur, laying down hidden dangers for vascular systolic and diastolic dysfunction, myocardial fibrosis, cardiac dysfunction, and even cardiac failure (5, 6). In this study, we found that: (1) BFRT and LMIRT effectively lowered the blood pressure of SHRs, improved the cardiac function of SHRs, reduce the thickness of the left ventricular wall of SHR, adjusted the ratio of collagen I to collagen III, reduced the collagen fiber total area, decreased the expression of TGFβ-1-Smad pathway-related proteins in the myocardium, and regulated the balance of MMP2/TIMP2 and MMP9/TIMP1. (2) HIRT significantly increased the BP of SHRs, especially the diastolic BP. Although it improved some cardiac functions of SHR, it did not reduce HR, LVPW, or the LV mass. Moreover, HIRT decreased the collagen I/III ratio and increased the expression of TGFβ-1-Smad pathway-related proteins in the myocardium, further decreasing the MMP/TIMP ratio. (3) The comparison between exercise groups found that BFRT was superior to traditional resistance training in lowering blood pressure and adjusting the proportion of collagen I and collagen III in the myocardium. The increase in the MMP2/TIMP2 ratio with BFRT was significantly higher than that with LMIRT, while the increase in the MMP9/TIMP1 ratio with LMIRT was significantly higher than that with BFRT.

In terms of cardiac morphology, function, and blood pressure, studies have shown that BFR exercise has a positive effect on the cardiac function and heart rate variability of healthy people, elderly people, and some patients with coronary artery disease, and improved the cardiac contractility and anti-ischemic ability, reduced heart rate and blood pressure, and increased heart rate variability (21–24). The same results were obtained in a study of elderly rats, in which BFR exercise improved cardiac function and arterial compliance (15, 25). However, the research on BFR exercise intervention in hypertension is mostly focused on an acute hypotensive effect, and there is a lack of research on the effect and mechanism of long-term BFRT on hypertension (26–29). Combined with the above studies, this study confirmed that 8-week BFRT effectively improved the cardiac function of early SHRs and lowered blood pressure. The results of LMIRT are consistent with previous studies, in which traditional low-intensity resistance training effectively lowered blood pressure and improved cardiac function (14). In addition, the antihypertensive effect of BFRT was better than that of LMIRT. Some studies have shown that exercise can reduce blood pressure by inhibiting sympathetic nerve excitability (30, 31), and other studies showed that the important influencing factors of blood pressure are cardiac output and peripheral resistance (32). Therefore, the better antihypertensive effect of BFRT is due to sympathetic nerve adaptation or the reduction of peripheral resistance, which needs further research to confirm. In regard to the HIRT results, some studies showed that high-intensity exercise caused an excessive pressure load of the heart, increased the deposition of collagen fibers, and produced adverse remodeling (33). Additionally, some researchers proposed that high-intensity exercise accelerated the redox dependent uncoupling of eNOS (34), increased the production of free radicals and oxidative stress, reduced the antioxidant capacity, and caused myocardial overload injury (35). When myocardial injury occurs, fibroblasts proliferate to repair wound healing (36). Moreover, when SHRs effectively correct the proportion of cardiovascular elastic collagen fibers, the aortic buffering function is promoted, and the myocardial elasticity is restored. The same stroke output no longer requires greater ventricular filling pressure, and the heart rate decreases (2, 10). However, the HR following HIRT did not decrease, and the BP was higher, which also showed that the underlying problem of SHR myocardial fibrosis in HIRT was not alleviated, and the intrinsic blood pumping required by the body was only achieved through the thickening of the myocardium and strengthening of the contractility. Naturally, high-intensity training also has a positive effect on cardiac function. It was reported that high-intensity training promoted the circulation of calcium in cardiac cells (37). It was also reported that myocardial cells completed effective pacing and performed regular contraction and relaxation under the effect of mitochondrial calcium ion dynamics, but the elimination of diastolic and systolic dysfunction and the short-term improvement of cardiac function cannot be taken as clinical evidence of cardiac function improvement. More evidence should be obtained from the comprehensive evaluation of changes in the composition of the extracellular matrix (including the increase and decrease in fibrosis), vascular sparsity, vascular dysfunction, and other factors (38, 39). In addition, some studies have proposed that high-intensity training increased sympathetic nerve excitability and caused adverse blood pressure fluctuations (40, 41). Combined with the above discussion, we believe that HLRT can improve some cardiac functions, but has adverse effects on BP and HR in SHRs.

In addition, the TGFβ-1-Smad pathway plays an important role in hypertensive myocardial fibrosis. It has been reported that when hypertensive myocardial fibrosis occurs, the TGFβ-1-Smad pathway in the myocardium is active, and TGFβ-1 and Smad2/3 are upregulated. When the TGFβ-1-Smad pathway is activated, Smad2 and Smad3 are phosphorylated, which can induce an increase in the CTGF expression level, and stimulate an increase in myocardial fibroblasts. At the same time, TGFβ-1 affects the downregulation of the MMP2/TIMP2 and MMP9/TIMP1 ratios, promotes an increase in collagen fiber synthesis, changes the proportion of collagen fiber types, and increases the overall collagen fiber content, thus triggering ventricular remodeling and leading to myocardial fibrosis (8, 9, 42). After effective intervention of drugs and exercise on myocardial fibrosis, the degree of myocardial fibrosis is reduced, the TGFβ-1-Smad pathway in the myocardium is inhibited, the expression level of CTGF is reduced, the balance between MMPs and TIMPs is adjusted, and the increase in collagen fiber deposition is avoided (14, 43). Although there are different opinions on the balance of MMPs and TIMPs during the development and treatment of myocardial fibrosis, it was found in early hypertensive models that MMPs decreased, TIMPs increased, and total collagen increased, which is consistent with an increase in myocardial fibrosis. These findings indicate that the difference in the expression of MMPs and extracellular matrix remodeling in response to pressure overload is a dynamic process, involving the accumulation and degradation of extracellular matrix, and depends on the stage of hypertension (42). The animal model of this study was early spontaneously hypertensive rats, and the results of BFRT and LMIRT in the experiment are also consistent with the above results. The experimental results of HIRT showed that the TGFβ-1-Smad pathway was activated, which further reduced the MMP/TIMP ratio and accelerated the synthesis of the extracellular matrix. Although HIRT reduced the collagen I/III ratio, the overall collagen fiber area in HIRT remained unchanged, indicating that the degree of myocardial fibrosis in SHR did not decrease. As for the results of HIRT, it was reported that high-intensity exercise induced hypertensive myocardial injury, leading to the formation of fibroblasts and affected cardiac function (44). Coincidentally, research by Rebelo et al. also showed that high-intensity training triggered renin-angiotensin system and induced upregulation of TGFβ-1 expression, resulting in adverse cardiac remodeling (45). Therefore, the control of resistance exercise intensity is also very important for the regulation of TGFβ-1-Smad pathway-related proteins in the myocardium of early spontaneously hypertensive rats. If the intensity is too high, the results run counter to the purpose of preventing and treating hypertension. In this regard, we believe that exercise can adjust the proportion of collagen fiber types in the early SHR myocardium, but the overall collagen fiber content is affected by exercise intensity.

Comparing the results of the collagen I/III ratio and MMP/TIMP balance regulation between the exercise groups, we think that the advantage of BFRT compared with traditional resistance training is blood flow restriction. Some studies have shown that blood flow restriction causes local laminar shear stress, which activates the mechanical conduction of endothelial cells, and stimulates the repair, migration, and proliferation of vascular endothelial cells. This also stimulated the production of NO, which helped repair the damaged endothelial cells of the heart muscle and protected the heart muscle through NO circulation. MMPs are partly derived from endothelial cells, and the increase in MMPs expression is conducive to the degradation of collagen fibers, and works together with TIMPs to regulate the proportion of collagen fibers (42, 46–48). MMP2 and MMP9 are both gelatinases. MMP2 can degrade a variety of collagen fibers in two steps, including collagen I and collagen III fibers. The collagen fibers that MMP9 can degrade do not contain collagen I and collagen III fibers. MMP2 is closely involved in several functions of endothelial cells. MMP2 increases the release of endothelium-derived hyperpolarization factor (EDHF) from endothelial cells. EDHF controls vasodilation, relieves vascular wall load, and reduces endothelial cell damage. Furthermore, overexpression of VEGFA released by endothelial cells increased the expression of MMP2 (42). Combined with the experimental results, we believe that BFRT may better repair endothelial cells in the SHR myocardium through blood flow restriction, improve endothelial cell dysfunction, release MMPs, degrade collagen, and adjust the ratio of collagen I to collagen III.

In this study, the effects of blood flow–restricted resistance training and traditional resistance training on myocardial fibrosis in early spontaneously hypertensive rats were compared and analyzed, and the blood flow restriction under exercise was observed. But there was a lack of research on the effect of blood flow restriction on myocardial fibrosis in early spontaneously hypertensive rats under simple quiet state. In this regard, the research team will conduct further research in the later stage.

In conclusion, blood flow restriction, combined with low and medium-intensity resistance training, inhibited the TGFβ-1-Smad pathway, reduced CTGF, regulated the balance of MMP2/TIMP2 and MMP9/TIMP1, and reduced the synthesis of collagen fibers, thereby reducing myocardial fibrosis and hypertrophy and improving cardiac function. BFRT also lowered blood pressure, achieved the preventive effect of early hypertension, and the hypotensive effect was better than traditional resistance training.

## Data availability statement

The original contributions presented in this study are included in the article/supplementary material, further inquiries can be directed to the corresponding author.

## Ethics statement

The animal study was reviewed and approved by the Animal Experiment Ethics Committee of the Nanjing Sport Institute.

## Author contributions

YZ devised the main conceptual ideas and designed the study with ZT. ZT performed the histological experiments with the supervision of YZ and the staining and western blot experiments, supervised the entire study, and edited the manuscript according to the guidance of YZ and PC. YCZ and YP participated in animal surgery, animal management, and helped in blood pressure recording and exercise training. BW participated in data analysis. All authors contributed to the article and approved the submitted version.
